# Do the Health Benefits of Cycling Outweigh the Risks?

**DOI:** 10.1289/ehp.0901747

**Published:** 2010-06-30

**Authors:** Jeroen Johan de Hartog, Hanna Boogaard, Hans Nijland, Gerard Hoek

**Affiliations:** 1 University of Utrecht, Institute for Risk Assessment Sciences, Utrecht, the Netherlands; 2 Netherlands Environmental Assessment Agency, Bilthoven, the Netherlands

**Keywords:** air pollution, biking, cycling, life table analysis, modal shift, physical activity, traffic accidents

## Abstract

**Background:**

Although from a societal point of view a modal shift from car to bicycle may have beneficial health effects due to decreased air pollution emissions, decreased greenhouse gas emissions, and increased levels of physical activity, shifts in individual adverse health effects such as higher exposure to air pollution and risk of a traffic accident may prevail.

**Objective:**

We describe whether the health benefits from the increased physical activity of a modal shift for urban commutes outweigh the health risks.

**Data sources and extraction:**

We have summarized the literature for air pollution, traffic accidents, and physical activity using systematic reviews supplemented with recent key studies.

**Data synthesis:**

We quantified the impact on all-cause mortality when 500,000 people would make a transition from car to bicycle for short trips on a daily basis in the Netherlands. We have expressed mortality impacts in life-years gained or lost, using life table calculations. For individuals who shift from car to bicycle, we estimated that beneficial effects of increased physical activity are substantially larger (3–14 months gained) than the potential mortality effect of increased inhaled air pollution doses (0.8–40 days lost) and the increase in traffic accidents (5–9 days lost). Societal benefits are even larger because of a modest reduction in air pollution and greenhouse gas emissions and traffic accidents.

**Conclusions:**

On average, the estimated health benefits of cycling were substantially larger than the risks relative to car driving for individuals shifting their mode of transport.

Recently, policy interest in promoting cycling as a mode of transport has increased substantially within Europe. Several capitals, such as Copenhagen, Denmark (in 1995), Helsinki, Finland (2000), Oslo, Norway (2002), Stockholm, Sweden (2006), Barcelona, Spain (2007), Paris, France (2007), and Brussels, Belgium (2009), have implemented low-cost rental systems aimed at stimulating commuters to use bicycles for the typically short urban trips. Motive for these policies is more often the reduction of traffic congestion than promotion of health. In 2005, the European Union formulated an important area of action: “addressing the obesogenic environment to stimulate physical activity” (Commission of the European Communities 2005). Attitudes and policies toward active commuting have recently been discussed (Lorenc et al. 2008; Ogilvie et al. 2004). The Transport, Health, and Environment Pan-European Programme (THE PEP) provides guidance to policy makers and local professionals on how to stimulate cycling and walking (THE PEP 2009). The promotion of walking and cycling is a promising way to increase physical activity across the population by integrating it into daily life.

Promoting cycling for health reasons implies that the health benefits of cycling should outweigh the risks of cycling. Although society may benefit from a shift from private car use to bicycle use (e.g., reduced air pollution emission), disadvantages to individuals may occur. Although individuals may benefit from increased physical activity, at the same time they inhale more pollutants because of increased breathing rates. The risks of being involved in traffic accidents may increase, as well as the severity of an accident. A study in Vancouver, Canada (Marshall et al. 2009), illustrated that, especially in the city center, high-walkability neighborhoods had high traffic density, leading to high air pollution concentrations for a traffic-related primary pollutant [nitric oxide (NO)] but not for a secondary pollutant (ozone). For cycling, similar issues may occur.

The aim of this review is to assess quantitatively whether the health benefits of the use of a bicycle instead of a private car for short trips outweigh the health risks. The risks and benefits are evaluated both for the individuals who shift from car driving to cycling and for society as a whole.

## Materials and Methods

We focus on the comparison of private car driving versus cycling because most trips are made by car, and the use of the private car is related to many negative aspects, including congestion, use of physical space, reduction of outdoor activities, air pollution, and noise. In the Netherlands, 20% and 30% of total car trips (totaling 15.9 million trips/day) are, respectively, for shopping and commuting purposes (Beckx et al. 2009a, 2009b; Mobiliteitsonderzoek Nederland 2007). Approximately 50% of all car trips are < 7.5 km, which is short enough to make travel by bicycle a feasible alternative.

In the quantitative comparison between car driving and cycling, we considered air pollution, traffic accidents, and physical activity as main exposures. We summarize the relevant evidence of health effects related to air pollution, traffic accidents, and physical activity separately. For these sections, we made use of published (systematic) reviews, supplemented with more recent key studies.

Health effects related to air pollution, traffic accidents, and physical activity differ—for example, traffic accidents resulting in injuries and physical activity affecting cardiovascular disease. Therefore, we compare potential effects of these exposures (in conjunction with driving or cycling) on mortality rather than morbidity. In addition, epidemiologic evidence of associations of these exposures with mortality is stronger than associations with other outcomes, particularly for physical activity. All three exposures have been associated with mortality, so a common metric can be used to quantify their potential effects, and mortality is reported more consistently than other health outcomes. In particular, minor injuries associated with traffic accidents are much more likely to be underreported than are deaths due to traffic accidents.

For deriving the relative risks comparing car driving and cycling, we specified a hypothetical scenario based on statistics in the Netherlands. The scenario assumes a transition from car driving to cycling for 500,000 people 18–64 years of age for short trips on a daily basis in the Netherlands. We made calculations for a daily traveled distance of 7.5 km and 15 km—for example, people commuting to and from work for 3.75 km (the average short trip) or 7.5 km (the maximum short trip). Our scenario implies a shift of about 12.5% of the 7.95 million short car trips, an ambitious yet not unrealistic percentage. In the Netherlands, 40.8% of persons > 18 years of age own both a car and a bicycle and therefore may be able to shift modes on a daily basis. In this review, we focus on the Dutch situation because of data availability, but in the overall discussion we illustrate that the use of this Dutch scenario has not substantially affected our conclusions. The scenario is used mostly to calculate travel time and kilometers driven, inputs needed to calculate air pollution, physical activity, and accident impacts, combined with more generic concentration–response functions.

We express mortality impacts in life-years gained or lost estimated with life table calculations (Miller and Hurley 2003). For the calculation we used a population of 500,000 people 18–64 years of age, distributed in age categories comparable to the 2008 Dutch population [Statistics Netherlands (CBS) 2008]. We estimated the effects on this population for a lifetime.

## Air Pollution Exposures and Health Effects

### Air pollution exposure during cycling and car driving

Since the 1990s various studies have measured air pollution exposure levels associated with different modes of transport (Kaur et al. 2007). In recent studies, the emphasis has been on fine and ultrafine particulate matter [aerodynamic diameter ≤ 2.5 μm (PM_2.5_) and ≤ 0.1 μm (UFP), respectively], because these are the main pollutants related to human health effects. Driving or cycling in traffic may result in air pollution exposures that are substantially higher than overall urban background concentrations (Kaur et al. 2007). Consequently, even relatively short times spent in traffic may contribute significantly to daily exposures (Beckx et al 2009a, 2009b; Fruin et al. 2004; Marshall et al. 2006; Van Roosbroeck et al. 2007). Table 1 summarizes studies that specifically compared exposures during car driving and cycling within the same study.

Overall, air pollution exposures experienced by car drivers were modestly higher than those experienced by cyclists, with mean ratios of 1.16 for PM_2.5_, 1.01 for UFP, and 1.65 for elemental carbon or soot. However, increased physical activity results in higher minute ventilation (volume of air inhaled in one minute) for cyclists than for car drivers, with estimates from two Dutch studies reporting that the minute ventilation of cyclists was 2.3 times (van Wijnen et al. 1995) and 2.1 times (Zuurbier et al. 2009) higher than that of car drivers. Therefore, inhaled doses of PM_2.5_ and, to a lesser extent, elemental carbon may be higher in cyclists. The difference in exposure between cyclists and car drivers depends on a large number of factors, such as selected route, car speed, trip duration, car type, ventilation status (open windows, mechanical ventilation), driving behavior, street configuration, and weather conditions (Kaur et al. 2007). Trip duration might also be higher for cyclists, although this may be highly dependent on the setting. For example, in a study conducted in 11 Dutch cities, there was no difference in the time required to bicycle versus drive short distances (Boogaard et al. 2009), but for longer trips cars were faster than cyclists (Zuurbier et al. 2010).

### Health effects of in-traffic exposures

The short exposures typical for commuting have not been studied extensively in air pollution epidemiology, in contrast to 24-hr average exposures or long-term (annual average) exposures [World Health Organization (WHO) 2006]. Several studies have documented that long-term exposure to traffic-related air pollution is associated with adverse health effects, including increased mortality (WHO 2006).

Table 2 summarizes the few epidemiologic studies of in-traffic air pollution exposures, suggesting that these exposures result in physiologic changes (including airway and systemic inflammation and lung function decrements) in healthy adults and asthmatics and possibly more severe adverse effects (myocardial infarction).

Furthermore, there is a fairly substantial body of evidence of human controlled exposure studies in which volunteers have been exposed for 1–2 hr to diesel exhaust and to filtered air for comparison [see Supplemental Material, Table 1 (doi:10.1289/ehp.0901747)]. Typically, the evaluated exposures (100–300 μg/m^3^) are higher than those encountered in ambient air, although not excessively. Because of ethical concerns, only physiologic effects have been studied with this study design. These studies have documented airway and systemic inflammation after exposure to diesel exhaust in patients and in healthy subjects.

### Assessment of the modal shift impact on mortality related to air pollution exposure

#### Individual effects

Because the physiologic changes observed in epidemiologic and controlled exposure studies likely play a role in the pathway to cardiac events of long-term exposure, it is plausible that these more adverse effects may occur in susceptible subjects. We calculated the potential impact on mortality of a transition from using a car to a bicycle for a 30-min (7.5-km) or 1-hr (15-km) commute based upon relative risk estimates from long-term exposure studies of mortality in association with PM_2.5_ (Pope et al. 2002) and black smoke (BS) (Beelen et al. 2008).

The derivation of these risk estimates is provided in the Supplemental Material, Table 2 (doi:10.1289/ehp.0901747); Table 3 shows the results. We assumed that the actual risk related to long-term air pollution exposure is determined by the inhaled daily dose of PM_2.5_ or BS. First, we calculated the inhaled pollution dose during commuting (car driving or cycling) and noncommuting hours based on prior information concerning minute ventilation rates (liters per minute) and PM_2.5_ and BS exposures (micrograms per cubic meter) during sleep, rest, driving, or cycling. Next, we estimated the total daily dose for PM_2.5_ and BS (micrograms per day) for driving or cycling. We then used the ratio of the total daily doses for the two travel modes to derive an “equivalent” change in PM_2.5_ or BS concentration (micrograms per cubic meter) that could be normalized to the 10-μg/m^3^ increase in long-term exposures used by Pope et al. (2002) and Beelen et al. (2008) to estimate the relative risk associated with the estimated change in long-term PM_2.5_ and BS exposures that would result from a shift to commuting by bicycle instead of by car.

Assuming equal toxicity of particles, the estimated relative risk associated with the change in PM_2.5_ inhalation due to cycling instead of car driving ranges from 1.005 to 1.010. If we assume that traffic PM is more toxic than ambient PM_2.5_ in general, these relative risk estimates range from 1.026 to 1.053. This assumption is supported by an analysis of PM from different sources, indicating the strongest associations with mortality from traffic particles (Laden et al. 2000). If the assessment is based on BS, relative risk estimates are smaller (between 1.001 and 1.012).

#### Societal effects

The modal shift will reduce overall air pollution levels, which may result in health benefits of the general city population. An indication of the potential reduction in air pollution was obtained by using the Dutch dispersion model CAR (Calculation of Air pollution from Road traffic) (Eerens et al. 1993). For a typical major urban street with a traffic intensity of 10,000 vehicles/day, for a 12.5% reduction in traffic intensity, concentration reductions were 1.3 μg/m^3^ for nitrogen dioxide (NO_2_) and 0.4 μg/m^3^ for particles with aerodynamic diameter ≤ 10 μm (PM_10_). The relative risk of long-term exposure to NO_2_ expressed per 10-μg/m^3^ increase on all-cause mortality is 1.10 (Tonne et al. 2008). This implies that for the approximately 800,000–1,600,000 subjects living in major streets in the Netherlands, mortality rates could be 1.012 times lower. This relative risk is of the same order of magnitude as the estimated increased risk to the cyclist described in the previous section and applies to a larger population.

## Accidents

According to the WHO (2004), road traffic injuries accounted for approximately 2% of all global deaths, making them the 11th leading cause of global deaths. The rates of road traffic death vary considerably among countries, transport mode, type of area (urban or rural), and person. Among several European countries, the highest fatality rates are about 3.5 times higher than the lowest figures [see Supplemental Material, Figure 1 (doi:10.1289/ehp.0901747)] (International Transport Forum 2010).

### How safe is cycling compared with car driving for an individual?

Table 4 shows the estimated numbers of traffic deaths per age category per billion passenger kilometers traveled by bicycle and by car (driver and passenger) in the Netherlands for 2008 (CBS 2008). These data suggest that there are about 5.5 times more traffic deaths per kilometer traveled by bicycle than by car for all ages, and that cycling is riskier than travel by car for all age groups except young adults (15–30 years of age), with about 9 times more deaths among those < 15 years of age, and 17 times more deaths among those > 80 years of age. The comparison in Table 4 probably overestimates the difference between cyclists and car drivers for short trips, because the relatively safe long car trips driven on highways are included. Across Europe, 8% of traffic deaths occur on the motorways, whereas 25% of the kilometers driven are on motorways (European Road Transport Safety 2008). Risks for nonfatal accidents are higher for cyclists than for car drivers, as well [Supplemental Material, Table 3 (doi:10.1289/ehp.0901747)].

### How safe is cycling compared with car driving for society?

For society, the risk that car drivers present to cyclists and pedestrians must also be taken into account. For the Netherlands, an analysis has compared the risks of a fatal accident for car drivers and cyclists, including the risk to other road users (Dekoster and Schollaert 1999). The analysis excluded motorways, because cyclists cannot use these roads. Mortality rates were similar for car drivers and cyclists (20.8 vs. 21.0 deaths per million kilometers traveled). People older than 50 years are less frequently involved in fatal accidents when driving a car than when driving a bicycle, but the opposite is true for people 18–49 years of age [Supplemental Material, Table 4 (doi:10.1289/ehp.0901747)]. Jacobsen (2003) showed that in different European countries, the number of traffic deaths of cyclists is inversely related to the amount of cycling [Supplemental Material, Figure 2 (doi:10.1289/ehp.0901747)], suggesting a “safety-in-numbers” effect.

### Assessment of the modal shift impact on traffic accidents related mortality

For 18- to 64-year-old individuals, the risk of a fatal accident while cycling is about 4.3 times higher compared with the same distance by car (Table 4). The fatal traffic accident rate for cyclists 20–70 years of age is about 8.2 deaths per billion passenger kilometers traveled, whereas the risk for car drivers and passengers the rate is 1.9 deaths per billion passenger kilometers traveled (Table 4). A population of 500,000 commuting 7.5 km/day will commute 1.36785 billion km/year (7.5 km/day × 365 days/year × 500,000). From the data shown in Table 4, we estimate that this amount of car travel would result in approximately 2.6 deaths/year (1.9 × 1.36785). An equivalent amount of bicycle travel would result in approximately 11.2 deaths/year (8.2 × 1.36785). In the Netherlands, the all-cause mortality rate for 18- to 64-year-old persons is 235.1 per 100,000 per year (CBS 2008) or 1,176 persons per 500,000 per year. Hence, among 18- to 64-year-olds, the relative risk of all-cause mortality associated with a 7.5 km/day shift from driving to cycling would be [1,176 + (11.2 – 2.6)]/1,176 = 1.007. When we use age-specific data, relative risks ranged from 0.996 to 1.010. For the 15-km scenario, age specific relative risks ranged from 0.993 to 1.020.

The societal impact of a modal switch on the number of fatal accidents largely depends on which people switch from car to bicycle. If it is the average population, the impact (including risk presented to other road users) would be practically zero [Supplemental Material, Table 4 (doi:10.1289/ehp.0901747)], but if young car drivers switched to a bicycle, it would decrease the number of fatal accidents. The opposite is true for elderly car drivers.

## Physical Activity

Levels of inactivity are high in virtually all developed and developing countries.a The WHO (2007a) estimates that 60–80% of the world’s population does not meet the recommendations required to induce health benefits. For Europe 62.4% inactive adults are estimated ranging from 43.3% (Sweden) to 87.7% (Portugal) (Varo et al. 2003). In the Netherlands about 62% of the population is sedentary (Varo et al. 2003). The WHO estimates that the prevalence of physical inactivity accounts for 22% of cardiovascular disease prevalence globally (WHO 2007a). There is sufficient evidence for an association between physical activity and mortality, cardiovascular disease (hypertension), diabetes, obesity, cancer (colon and breast), osteoporosis, and depression (Bauman 2004; Warburton et al. 2006). Because only a few studies specifically reported on the beneficial health effects of cycling, we also summarized the quantitative evidence of beneficial health effects of physical activity, making use of review papers.

### Cycling and physical activity recommendation

Recently, the American College of Sports Medicine and the American Heart Association published an updated recommendation for physical activity (Haskell et al. 2007). To promote and maintain health, all healthy adults 18–65 years of age need moderate-intensity aerobic physical activity for a minimum of 30 min on 5 days each week or vigorous-intensity aerobic activity for a minimum of 20 min on 3 days each week. Also, combinations of moderate- and vigorous-intensity activity can be performed to meet this recommendation. For young people, 60 min of moderate to vigorous physical activity on a daily basis is recommended (Strong et al. 2005). In several physical activity studies, metabolic equivalent of task (MET) is used as an indicator of physical activity, and the minimum goal should be in the range of 500–1,000 MET min/week. Leisure cycling or cycling to work (15 km/hr) has a MET value of 4 and is characterized as a moderate activity (Ainsworth et al. 2000). Hence, a person shifting from car to bicycle for a daily short distance of 7.5 km would meet the minimum recommendation (7.5 km at 15 km/hr = 30 min) for physical activity in 5 days (4 MET × 30 min × 5 days = 600 MET min/week).

### Health effects and assessment of the modal shift impact on mortality

Table 5 provides summary estimates from reviews for the impact of physical activity on all-cause mortality, and includes only estimates that are relevant to compare the risks for cyclists and car drivers. It is difficult to synthesize information across studies because investigators have measured physical activity in different ways and classified physical activity according to different dose schemes that often are difficult to compare directly (Lee and Skerrett 2001). Several reviews have suggested that the relative risk of mortality for those who meet the recommended levels of physical activity compared with the inactive group is between 0.65 and 0.80 (Bauman 2004; Lee and Skerrett 2001; Warburton et al. 2006).

Three studies have directly assessed mortality related to cycling to work. In a prospective study in Copenhagen, the relative risk of the group bicycling to work was 0.72 [95% confidence interval (CI), 0.57–0.91] compared with other modes of transport after multivariate adjustment, including leisure-time physical activity (Andersen et al. 2000). The relative risk for physically active groups compared with the sedentary group decreased with activity level: 0.68, 0.61, and 0.53 (Andersen et al. 2000). In the Shanghai Women’s Health Study, exercise and cycling for transportation were both inversely and independently associated with all-cause mortality (Matthews et al. 2007). Hazard ratios were 0.79 (95% CI, 0.61–1.01) for the group cycling 0.1–3.4 metabolic equivalent hours per day and 0.66 (95% CI, 0.40–1.07) for the group cycling > 3.4 metabolic equivalent hours per day, compared with the noncycling group. A Finnish study that combined cycling and walking to work versus nonactive commuting also showed significantly lower relative risks for active commuters in the range of 0.71 and 0.79 (Hu et al. 2004). According to the reviews and the three cycling studies, the relative risk for all-cause mortality is in the range of 0.50–0.90 (Table 5).

An expert panel determined a generally linear relationship between physical activity level and the rates of all-cause mortality, total cardiovascular disease, and coronary heart disease incidence and mortality (Kesaniemi et al. 2001). There is thus evidence that health gains occur for physically active and nonactive persons, although the magnitude of these benefits may differ.

To calculate the potential impact of the modal shift on mortality, we directly used the range of relative risk estimates (0.50–0.90) presented in Table 5.

## Comparison of Life Years Gained or Lost

For the people who shift from car to bicycle use for short trips, we estimated that the beneficial effect on all-cause mortality rates of the increased physical activity due to cycling is substantially larger (relative risk, 0.50–0.90) than the potential mortality effect of increased inhaled air pollution doses (relative risk, 1.001–1.053) and the effect on traffic accidents (age-specific relative risk, 0.993–1.020). The estimated gain in life expectancy per person from an increase in physical activity ranged from 3 to 14 months (Table 6). The estimated life expectancy lost because of air pollution (0.8–40 days) and traffic accidents (5–9 days) was much smaller. On average, the benefits of cycling were about 9 times larger than the risks of cycling, compared with car driving for the individuals making the shift, calculated as 337,896/(28,135 + 9,639). The estimated number of life years gained still exceeded the losses when the lowest estimate for physical activity was compared with the highest estimate for air pollution and traffic accidents (benefits/risks ratio of 2).

The largest estimated gain in life years was for the elderly [Supplemental Material, Table 6 (doi:10.1289/ehp.0901747)]. The ratio of life years gained to lost was 8.4 for persons < 40 years of age, 8.6 for persons 40–64 years of age, and 10.8 for persons ≥ 65 years of age.

The relative benefits of a 7.5-km versus 15-km distance are probably similar. A 15-km distance (1-hr commute) increases the life-years lost for air pollution from 20 to 40 days based on PM_2.5_ and increases the life-years lost for traffic accidents from 5 to 9 days. The total estimated number of days lost per person is thus 49 for a 15-km distance and 25 for a 7.5-km distance. The relative risk of physical activity is difficult to quantify with the approach employed here. Using the data from Matthews et al. (2007), the relative risk would be 0.79 for the 7.5-km distance and 0.66 for the 15-km distance, assuming 4 MET associated with cycling. These relative risks translate in 280 and 173 days gained, respectively.

## Overall Discussion

### Principal findings

We quantitatively compared the health benefits from physical activity with the risks related to air pollution and traffic accidents between cycling and car driving for short trips, distinguishing the individuals who shift modes of travel from society as a whole. Estimated inhaled air pollution doses were higher in cyclists. The risk of a fatal traffic accident is higher for cyclists than for car drivers. Substantial benefits of physical activity have been demonstrated, including decreased cardiovascular disease and mortality.

For the people who shift from car to bicycle, we estimated that the well-documented beneficial effect of increased physical activity due to cycling resulted in about 9 times more gains in life-years than the losses in life years due to increased inhaled air pollution doses and traffic accidents. For the society as a whole this can be even larger because of reduced air pollution emissions. If the risk presented to other road users is included, the risk of a fatal traffic accident is virtually the same for cyclists and car drivers.

### Strengths and weaknesses

The strength of our assessment is especially the quantitative comparison of benefits and risks, in a common scenario for the three stressors evaluated. It could be argued that the Copenhagen (Andersen et al. 2000) and Chinese studies (Matthews et al. 2007) of the effects of bicycling on mortality have already demonstrated the net effect of physical activity on all-cause mortality, including the negative effects of fatal traffic accidents and air pollution. However, the size of the potential negative health effects was not quantified separately in those studies. Therefore it is difficult to transfer the net effect of these studies to other locations, where traffic accident rates and air pollution may be different. Because in our assessment the separate risks have been disentangled, it is possible to make assessments for different settings, by using other input data (e.g., traffic mortality rates).

We performed our calculations for the Netherlands, where an extensive cycling infrastructure exists and priority is given to cyclists over other traffic—factors that contribute to regular cycling. Restrictions to car use through traffic calming in residential areas and car-free zones influence cycling behavior as well (Pucher and Dijkstra 2003). Apart from the highest average distance cycled per person, the Netherlands is also one of the safest countries in terms of fatal traffic accidents. In such countries as the United Kingdom, Spain, and France, the risk of a fatal traffic accident for cyclists is substantially higher, probably also relative to car driving [Supplemental Material, Figure 2 (doi:10.1289/ehp.0901747)]. When we repeated the traffic accident calculations for the United Kingdom, where the risk of dying per 100 million km for a cyclist is about 2.5 times higher [Supplemental Material, Figure 2 (doi:10.1289/ehp.0901747)] and assuming the same fatality risk for car drivers as in the Netherlands, resulting life expectancy losses were approximately 14 days/person, based on 2005 population data from the United Kingdom and Wales. Overall, benefits of cycling are still 7 times larger than the risks.

Calculations on mortality impacts were performed for people 18–64 years of age, because people in that age range are more likely to make the modal shift. Age-specific analysis showed that the relative benefits of cycling are highest in the older age categories. This may have been even more pronounced if we had taken into account that the relative risks of physical activity may be larger for the elderly (Vogel et al. 2009). The empirical evidence for higher relative risks in elderly related to long-term exposure to air pollution is weak; for example, in the large American Cancer Society study there were no differences in relative risk for PM_2.5_ (Pope et al. 2002). We did not include children in our assessment because they are unable to drive a car, so a modal shift is not possible. Because of our focus on mortality effects (being extremely rare in children), we could not quantitatively compare risks for children as car passengers or as cyclists for physical activity and air pollution. The benefits of physical activity in children are considered important, however, both for current and for future health.

Overall relative risks may largely reflect the response from sensitive subgroups. For all stressors, the elderly are likely more susceptible, and we documented in an additional analysis that the ratio of benefits and risks was highest for ≥ 65-year-olds. For air pollution, subjects with preexisting cardiorespiratory disease may be more susceptible, and for physical activity, sedentary people may be more susceptible; these are subgroups that may partly overlap. Hence, both the risks and benefits may be higher than in the population average analysis.

In summary, it is unlikely that the conclusion of substantially larger benefits from cycling than risks is strongly affected by the assumptions made in the scenario, including the use of data from the Netherlands. Because concentration–response functions are mostly based on studies in Europe and North America, they may not apply in developing countries. For air pollution, there are no studies on long-term mortality effects in developing countries. The generally higher ambient air pollution concentrations could lead to higher losses in life-years comparing cycling and car driving. Traffic accident statistics for the Netherlands are probably not transferable to developing countries. For physical activity, there is evidence from a Chinese study (Matthews et al. 2007), with very similar benefits. Hence, very large differences in concentration–response functions for air pollution and traffic accidents from the functions we used would be necessary to tip the balance between benefits and risks.

For air pollution, there is considerable evidence that long-term and short-term exposures are related to increased cardiopulmonary mortality (Brunekreef and Holgate 2002). There are no studies of mortality effects specifically related to in-traffic exposures. We estimated the effect of shifting mode using two major long-term mortality cohort studies (Beelen et al. 2008; Pope et al. 2002), making assumptions about the contribution of traffic participation to the total inhaled dose of PM_2.5_ and (diesel) soot. Relative risks comparing cycling and car driving were small for both approaches, with the lower estimates based upon BS probably most realistic, because this component is more specific for traffic emissions.

The actual risk may be smaller because cyclists could more easily choose a low-traffic route. The substantial influence of route has been documented in various monitoring and modeling studies (Adams et al. 2001; Hertel et al. 2008; Kingham et al. 1998; Strak et al. 2010). A study in Utrecht found 59% higher UFP exposure for cyclists along a high-traffic route compared with a low-traffic route (Strak et al. 2010). Walking close to the curb in London greatly increased personal exposures (Kaur et al. 2005). For cyclists, position on the road is likely important as well, because it determines distance to motorized traffic emissions. Urban planning may also contribute by separating cycle lanes from heavily trafficked roads (Thai et al. 2008).

For society, reduced overall air pollution levels may result in lower mortality from long-term exposure of city dwellers. The potential benefits we estimated based on NO_2_ reductions were in the same order of magnitude as the potential risks for the individuals shifting.

Table 4 shows that the modal shift will lead to an increase in traffic accident deaths. The relative risk may be lower than we used because of the “safety-in-numbers” effect [Supplemental Material, Figure 2 (doi:10.1289/ehp.0901747)]. Car drivers may take more account of cyclists, resulting in fewer accidents per car-kilometer, when cyclists form a bigger part of the traffic (Jacobsen 2003). Traffic fatality and injury rates in Germany and the Netherlands (with relatively high levels of cycling and walking) were relatively low compared with those of the United States (Pucher and Dijkstra 2003). However, whether this reduction is attributable to a safety-in-numbers effect or a result of more biking lanes cannot easily be disentangled. The WHO concluded that if promotion of active commuting is accompanied by suitable transport planning and safety measures, active commuters are likely to benefit from the safety-in-numbers effect (WHO 2007b). The relative risks could also be higher because the less experienced cyclists making the shift could be more vulnerable to accidents. We cannot quantify this effect.

Even when origin and destination are the same, cars and bicycles often take different routes (Witlox 2007). The same short trip for a car may be 20–50% longer than for a bicycle (our calculations are based on comparisons per kilometer). If we could make a trip-based comparison, a lower relative risk for fatal accidents for cyclists compared with car drivers would be found. Furthermore, we did not take into account the concept of constant travel time budgets (van Wee et al. 2006): A change of travel time will be compensated by a change of destination. When taking the bicycle, the shop next door is preferred over the shop with greater choices farther away. These factors would lead to lower relative risks than we used.

Relative risks for different physical activity definitions (total physical activity, meeting the physical activity guideline, active commuting) were quite consistent. An important issue is whether the comparison between subjects with lower and higher physical activity can be used to assess the health effects of a change in physical activity related to a shift toward active commuting. Bauman (2004) showed that persons who were already in the highest quartile of fitness at baseline had a significantly lower mortality when they became even more active. In another study, people who went from unfit to fit over a 5-year period had 44% relative risk reduction compared with people who remained unfit (Blair et al. 1995). The largest improvements in health status are seen in inactive persons who change their lifestyle and become physically active (Warburton et al. 2006). A review by Erikssen et al. (1998) suggested similar health benefits from an increase in physical activity for active and sedentary persons. Already active persons could have lower benefits of the extra physical activity, leading to relative risks up to 0.90. If only active persons shift mode of transport, lower overall benefits of cycling compared with car driving will be found (ratio of life-years gained vs. lost, 4 instead of 9).

An increase in cycling does not necessarily lead to an increase in total physical activity, if it is associated with reduced activity in another domain (Forsyth et al. 2008; Thomson et al. 2008). The empirical evidence for substitution is weak, and increased fitness could also lead to more physical activity in leisure time. If we assume that for 25% of the population no health gains occur because of substitution, the ratio of benefits to risks (central estimates from Table 6) would be reduced from 8.9 to 6.7. Only if for 89% of the population no increase in total physical activity occurs because of substitution would benefits and risk become equal.

We have not considered the negative effects of physical activity on health—namely, musculoskeletal injury and fatal and nonfatal cardiac events (Institute of Medicine 2007). Cycling can be considered a moderate type and duration of sport and has lower injury risk than do more vigorous types (running, scholastic athletics) and longer durations of physical activity (Hootman et al. 2001; Parkkari et al. 2004). Exercise has acute cardiac risks as well, but the absolute risk of a cardiac event during exercise seems to be low (Institute of Medicine 2007). Regular physical activity also reduces the acute risk of a cardiovascular event (Tofler and Muller 2006).

### Restriction to mortality

We limited the quantitative assessment to mortality. It is difficult to evaluate the comparison between cycling and car driving if morbidity is included because of the lack of solid concentration–response relationships for air pollution and physical activity for morbidity outcomes. A meta-analysis reported a consistent positive association between physical activity and health-related quality of life (Bize et al. 2007). The largest cross-sectional study showed that people meeting the recommended levels of physical activity had an adjusted odds ratio of “having 14 or more unhealthy days during the previous months” of 0.4 (95% CI, 0.36–0.45) over the inactive subjects (Bize et al. 2007). Quality of life may even further improve apart from the increases in life-years. Concentration–response functions for air pollutants and morbidity outcomes such as hospital admissions are lower than for mortality: in the range of 1% compared with 6% per 10-μg/m^3^ increase in PM_2.5_ (WHO 2006). Traffic injuries may differ even more between cyclists and car drivers than fatal accidents [Supplemental Material, Table 4 (doi:10.1289/ehp.0901747)], if underreporting of especially cyclist accidents is accounted for. This would reduce the ratio between benefits and risks.

We did not include all stressors in the quantitative evaluation. Cycling contributes to other benefits, including reduced emissions of carbon dioxide relevant for reducing climate change, reduced use of physical space (e.g., related to parking), and reduced traffic noise for city dwellers, which may result in less annoyance. We are not aware of exposure studies or health effects studies that have compared traffic noise during transport for cyclists and car drivers.

### Suggestions for policy

Our study suggests that policies stimulating cycling likely have net beneficial effects on public health. Policies should be accompanied by safety measures and efforts to limit hazards, for example, by infrastructural choices (building cycling lanes away from major roads to limit cyclists’ air pollution exposures) or limitations such as a ban on car traffic during school start and end hours near schools. Policies may take the age dependence of the traffic accident relative risks into account—for example, by stimulating especially the young to increase cycling. However, this may not be the optimal choice for the beneficial effects of cycling.

Assessing what traffic policies are effective in promoting a population shift from using cars toward cycling (and walking) is beyond the scope of this review. A recent review showed that targeted behavior change programs can change the behavior of motivated subgroups, resulting in a 5% shift of all trips at the population level in the largest study (Ogilvie et al. 2004). However, effects of similar programs on the general, less motivated population are unclear. Those programs may benefit from taking the public’s views into account and learning from good practices (e.g., THE PEP 2009). In particular, perceptions of walking and cycling as dangerous activities are an important barrier to the promotion of active transport (Lorenc et al. 2008).

## Conclusions

On average, the estimated health benefits of cycling were substantially larger than the risks of cycling relative to car driving. For the society as a whole, this can be even larger because there will be a reduction in air pollution emissions and eventually fewer traffic accidents. Policies stimulating cycling are likely to have net beneficial effects on public health, especially if accompanied by suitable transport planning and safety measures.

## Figures and Tables

**Table 1:** Air pollution exposures during cycling and car driving.

City	Study design	Pollutant	Mean concentration car (μg/m^3^)	Mean concentration cycling (μg/m^3^)	Ratio car/cycle	Reference
Amsterdam	Two inner-city routes traveled for about 1 hr in January and May 1990 (*n* = 55 and 41)	CO BTEX	4,833 332	1,730 99	2.8 3.4	van Wijnen et al. 1995
Copenhagen	Two cars and two cyclists on a 7.6-km inner-city route in the morning of two days in summer 1998	BTEX TSP	44 44	150 75	0.3 0.6	Rank et al. 2001
London	Three routes from the center (one central, two to more outward sections) in July 1999 and February 2000 (*n* = 96 cycle trips and 54 car trips)	PM_2.5_ EC	37 29	28 18	1.32 1.6	Adams et al. 2001
London	Two short (~ 1 km) routes (one heavy traffic, one mixed) traveled in spring 2003 during early morning, lunchtime, and afternoon	EC	39	25	1.6	Gegisian 2003
London	Two short (~ 1 km) routes (one heavy traffic, one mixed) traveled in spring 2003 during early morning, lunchtime, and afternoon	PM_2.5_ UFP CO	38 99,736 1,300	34 93,968 1,100	1.12 1.06 1.18	Kaur et al. 2005
Huddersfield, UK	7-mile journey from village to Huddersfield, cycle along a major highway and a separate bicycle path (six samples in September/October 1996)	Abs	7.6	2.7 6.3	2.6 1.2	Kingham et al. 1998
11 Dutch cities	Simultaneous cycle and car drives between same start and end points in afternoon in 11 large Dutch cities, ~ 12 routes in each city; sampling duration, ~ 3 hr/city (1 day per city in autumn 2006)	UFP PM_2.5_	25,545 49	24,329 45	1.05 1.11	Boogaard et al. 2009
Arnhem, the Netherlands	2-hr morning rush hour exposures of cyclists and car and bus passengers on an urban route in a medium-size city	UFP PM_2.5_ Abs	40,351 78 8.8	44,258 72 6.0	0.91 1.09 1.48	Zuurbier et al. 2010
Mean	Simple mean of ratios from applicable studies	PM_2.5_ EC and Abs UFP			1.16 1.65 1.01	

Abbreviations: Abs, absorbance (10^−5^ m), a marker for (diesel) soot; BTEX, sum of benzene, toluene, ethylbenzene and xylene; CO, carbon monoxide; EC, elemental carbon, equivalent to (diesel) soot; TSP, total suspended dust; UFP, ultrafine particle count (per cubic centimeter).

**Table 2:** Epidemiological studies of air pollution exposure in traffic.

Study population	Design	Main findings	Comments	Reference
Sixty mild to moderate asthmatic adults in London	Exposure during 2 hr walking in OS or HP, pre/postexposure physiologic measurements: median PM_2.5_ concentration, 28 (OS) vs. 11 μg/m^3^ (HP); median EC, 7.5 vs. 1.3 μg/m^3^; median UFP, 63,700 vs. 18,300 particles/cm^3^	Asymptomatic decrease in lung function and increase in inflammation after walking in OS compared with HP; changes most consistently associated with EC and UFP; per 1-μg/m^3^ significant increase in EC decrement in lung function of ~ 1% decrement in lung function and ~ 2% increase in exhaled NO (inflammation)	OS has diesel traffic only	McCreanor et al. 2007
Subjects (*n* = 691) with MI in Augsburg	Case–crossover study comparing the frequency of participation in traffic in the hours before the MI and a control period (24–72 hr before MI)	RR = 2.92 for participation in traffic in the hour before the MI; increased risk found for all transport means (car, bicycle, public transport)	May be stressors other than air pollution	Peters et al. 2004
Nine healthy young U.S. policemen	Physiologic measurements before and after 8-hr work shift; average in-vehicle PM_2.5_, 24 μg/m^3^	Significant increases of heart rate variability, ectopic beats, blood inflammatory and coagulation markers, and red blood cell volume; per 10-μg/m^3^ PM_2.5_ effect on C-reactive protein, +32%; neutrophils, +6%; von Willebrand factor, +12%; and ectopic beats, +20%.		Riediker et al. 2004
Twelve healthy young subjects	Physiologic measurements before and after 1-hr cycling trip from city center to university in Utrecht	Statistically nonsignificant 1–3% decrements in lung function per 10^5^/m soot concentration and a 15% increase in exhaled NO per 38,000 particles/cm^3^		Strak et al. 2010

Abbreviations: EC, elemental carbon; HP, Hyde Park; MI, myocardial infarction; NO, nitric oxide; OS, Oxford Street; RR, relative risk; UFP, ultrafine particle count.

**Table 3:** Potential mortality impact of cycling compared with car driving, for 0.5- and 1-hr commute, estimated for PM_2.5_ and BS.^a^

Travel mode	Duration of travel (hr/day)	PM_2.5_/BS concentration (μg/m^3^)	Inhaled dose (μg/day)	Total dose^b^ for car or bicycle (μg/day)	Equivalent change in PM_2.5_ or BS (μg/m^3^)	RR mortality, equal toxicity^c^	RR mortality, traffic 5× more toxic
PM_2.5_							
Car	0.5	40.0	12.0	246			
Cycle	0.5	34.5	22.8	257	0.9	1.005	1.026
Car	1.0	40.0	24.0	252			
Cycle	1.0	34.5	45.5	274	1.8	1.010	1.053

BS							
Car	0.5	30.0	9.0	126			
Cycle	0.5	18.2	12.0	129	0.2	1.001	1.006
Car	1.0	30.0	18.0	132			
Cycle	1.0	18.2	24.0	138	0.5	1.002	1.012

RR, relative risk.

a Supplemental Material, Table 2 (doi:10.1289/ehp.0901747), gives details on calculations and assumptions.

b Total dose includes other time periods.

c RR for cycling versus car driving.

**Table 4:** Traffic deaths per age category per billion passenger kilometers by bicycle and by car in the Netherlands.^a^

Age category (years)	Bicycle	Car	Ratio
< 15	4.9	0.6	8.6
15–20	5.4	7.4	0.7
20–30	4.2	4.6	0.9
30–40	3.9	2.0	2.0
40–50	6.6	1.0	6.9
50–60	9.6	1.2	7.9
60–70	18.6	1.6	11.7
70–80	117.6	7.6	15.4
> 80	139.6	8.1	17.1
Total average (all ages)	12.2	2.2	5.5
Total average (20–70 years of age)	8.2	1.9	4.3

Data from CBS (2008).

a Estimated as age-specific and traffic mode–specific number of traffic deaths divided by amount of kilometers driven per age and traffic mode in the Netherlands for the year 2008.

**Table 5:** Potential impact of physical activity on all-cause mortality in various reviews^a^ and cohort studies.

Source	Definition of physical activity	Relative risk^b^	Comments
Reviews			
Lee and Skerrett 2001	Meeting moderate physical activity recommendation (1,000 kcal/week)	0.70–0.80	Review, excluding papers examining only two levels of physical activity
Kesaniemi et al. 2001	Expending of 1,000 kcal/week	0.70	Based on a symposium; invited experts reviewed the existing literature
Bauman 2004	Meeting physical activity recommendation	0.70	Review of peer-reviewed studies published between 2000 and 2003
Bucksch and Schlicht 2006	Different definitions of physical activity	0.70–0.87 (moderate) 0.46–0.92 (vigorous)	Review
Warburton et al. 2006	Meeting physical activity recommendation	0.65–0.80	Review
Vogel et al. 2009	Different definitions including moderate exercise (4,100–7,908 kJ/week), vigorous exercise, and different distances walked	0.50–0.77	Review of adult cohort studies with a mean > 60 years of age

Studies on cycling			
Andersen et al. 2000	Cycling to work for 3 hr/week	0.55–0.72	Based on a Danish cohort, adjusted for leisure time physical activity (among others)
Hu et al. 2004	Walking and cycling to work	0.71–0.79	Based on a Finnish cohort study among subjects with type 2 diabetes; estimates without adjusting for other domains in physical activity
Matthews et al. 2007	Cycling to work (MET-hours/day)	0.66–0.79	Based on a Chinese women cohort in Shanghai, adjusted for other physical activity
Overall summary		0.50–0.90	

a Reviews used are often overlapping (reviewing the same evidence).

b Comparing physically active with physically less active.

**Table 6:** Summary of impact on all-cause mortality for subjects shifting from car to bicycle.

Stressor	Relative risk	Gain in life years^a^	Gain in life days/months per person^a^
Air pollution	1.001 to 1.053	−1,106 to −55,163 (−28,135)	−0.8 to −40 days (−21 days)
Traffic accidents	0.996 to 1.010^b^ 0.993 to 1.020^b^	−6,422 to −12,856 (−9,639)	−5 to −9 days (−7 days)
Physical activity	0.500 to 0.900	564,764 to 111,027 (337,896)	14 to 3 months (8 months)

a Applied to the 500,000 subjects 18–64 years of age making the shift, with standard life table calculations (Miller and Hurley 2003). Numbers in parentheses are the averages of the life gains (a minus sign indicates a loss of life years).

b We have applied age group–specific relative risks in the life table calculations; for the range, see Supplemental Material, Table 5 (doi:10.1289/ehp.0901747). The 0.996 to 1.010 figure is for the 7.5-km distance, and 0.993 to 1.020 is for the 15-km distance.
